# The Effect of Granulometry on the Flexural Behavior of Epoxy/*Washingtonia robusta* Particulate Biocomposites from Concón, Chile

**DOI:** 10.3390/polym18172050

**Published:** 2026-08-24

**Authors:** Héctor Michael Solar Cortés, María Elena Fernández Abreu, José Luis Valin Rivera, Meylí Valin Fernández, Daniel Francisco Leiva Palomera, Roberto Iquilio Abarzúa, Gilberto Garcia del Pino

**Affiliations:** 1 Escuela de Ingeniería Mecánica, Pontificia Universidad Católica de Valparaíso, Valparaíso 2430000, Chile; hector.solar.c@mail.pucv.cl (H.M.S.C.); maria.fernandez.a@pucv.cl (M.E.F.A.); daniel.leiva.p@mail.pucv.cl (D.F.L.P.); roberto.iquilio@pucv.cl (R.I.A.); 2 Department of Mechanical Engineering (DIM), Faculty of Engineering (FI), University of Concepción, Concepción 4070409, Chile; 3 Departamento de Engenharia Mecânica, Universidade do Estado do Amazonas (UEA), Manaus 69050-020, Amazonas, Brazil; gpino@uea.edu.br

**Keywords:** epoxy biocomposites, particulate reinforcement, natural fiber composites, *Washingtonia robusta*

## Abstract

Ornamental palm pruning residues represent a locally abundant, underutilized lignocellulosic waste stream with potential as a waste-valorized epoxy reinforcement. This study investigates the flexural behavior of particulate epoxy composites reinforced with *Washingtonia robusta* leaf stalk residue, evaluating the influence of reinforcement granulometry on mechanical and microstructural response. Four specimen families were fabricated from a Bisphenol A/F epoxy resin cured with a cycloaliphatic amine hardener: neat resin (RS, reference) and composites reinforced with fine (RF), coarse (RG) and mixed-fraction (RM) particles at 20 vol.% loading. Flexural properties were assessed by three-point bending and fracture surfaces were characterized by SEM. The neat resin exhibited a non-monotonic, viscoelastic-dominated response with no fracture within the extended deformation range tested, whereas all reinforced systems fractured within a substantially narrower window (~8–14.5 mm). RF showed the highest observed flexural modulus (≈15.8 GPa), followed by RM (≈15.4 GPa) and RG (≈14.2 GPa). These differences were not statistically significant (one-way ANOVA, *p* > 0.05). Damage tolerance followed a similar descriptive trend: RG failed earliest, linked to large interfacial pull-out cavities; RF delayed fracture through crack deflection; and RM showed the most favorable overall balance, combining a modulus comparable to RF with superior crack path tortuosity. These results indicate the potential of *Washingtonia robusta*, particularly in mixed-granulometry form, as a candidate reinforcement for semi-structural epoxy biocomposites, pending further characterization of properties such as tensile strength, impact resistance, moisture absorption, and long-term durability.

## 1. Introduction

Over the last decade, the polymer composite industry has been under increasing pressure to reduce its reliance on petroleum-derived reinforcements and to adopt renewable, low-carbon-footprint alternatives without sacrificing mechanical performance [1,2,3,4]. This shift is driven jointly by regulatory pressure on plastic waste and the rising cost and environmental impact of glass and carbon fibers. It is also driven by a growing scientific consensus that lignocellulosic residues, agricultural, forestry and urban pruning waste represent an underexploited resource base for structural and semi-structural materials [1,3,5]. Within this context, particulate and short-fiber biocomposites based on thermosetting matrices have attracted particular attention because they combine straightforward, low-cost manufacturing routes with the possibility of tailoring mechanical response through reinforcement content, geometry and surface characteristics [2,4,6].

Natural fiber-reinforced epoxy composites (NFRECs) have been the subject of extensive review in recent years, with jute, sisal, hemp, flax, kenaf, bagasse and date palm among the most widely studied reinforcements [1,3,4,7]. Epoxy resins are preferred as a matrix for these systems owing to their low cure shrinkage, high mechanical strength, excellent chemical and moisture resistance and compatibility with ambient or moderate-temperature curing schedules, which makes them attractive for small-batch and semi-industrial biocomposite fabrication [4,5,8]. At the same time, these reviews consistently identify the fiber–matrix interface as the single most influential parameter governing stress transfer from the polymer matrix to the reinforcement. It is therefore the primary lever for engineering the mechanical behavior of the composite [2,3,7].

Beyond continuous or long-fiber architectures, particulate lignocellulosic reinforcement has emerged as a practical alternative when the raw material is only available as short, irregular residues, as is the case for many agricultural and ornamental plant byproducts [1,2,6,8]. Particulate epoxy composites offer manufacturing advantages: simple mixing, casting, and compression molding routes. Their performance, however, is highly sensitive to processing variables such as compaction pressure, curing temperature profile, and post-cure schedule. These variables influence the final porosity and the degree of resin impregnation around the particles [2,4,5].

Among the processing variables specific to particulate systems, particle size and particle loading have repeatedly been shown to exert a decisive influence on stiffness, strength and toughness. Reviews of particulate organic filler polymer composites report that finer particle fractions, provided the critical aspect ratio is maintained, tend to improve stress transfer and mechanical properties relative to coarser fractions. While excessive filler loading beyond an optimum threshold promotes agglomeration, void formation and a corresponding drop in flexural and tensile performance [1,2,6,7]. This granulometric sensitivity is particularly relevant for residues obtained from non-engineered biomass sources, where particle size distribution is inherently heterogeneous and difficult to standardize compared with commercial synthetic fillers [2,3].

Microstructural characterization by scanning electron microscopy (SEM), frequently complemented by energy-dispersive X-ray spectroscopy (EDX), has become a standard tool for explaining the mechanical trends observed in flexural and tensile testing of particulate and short-fiber biocomposites. Fractographic studies consistently report mechanisms such as particle pull-out, interfacial debonding, crack deflection around rigid particles and localized matrix micro-cracking as the dominant damage modes controlling flexural failure in these systems [2,3,4,9]. These fracture mechanisms are directly linked to the quality of interfacial adhesion. Adhesion quality, in turn, depends on the chemical compatibility between the hydrophilic lignocellulosic surface and the hydrophobic epoxy matrix, a mismatch well documented across natural fiber and natural particle epoxy systems [3,5,8].

Palm-derived residues in particular have been investigated as epoxy reinforcement, most notably from date palm (*Phoenix dactylifera*) leaves, petioles and branches with reported flexural strengths that are strongly dependent on fiber/particle content, treatment, and manufacturing route [5,6,8,9]. However, the overwhelming majority of this literature is concentrated on a narrow set of palm species and traditional natural fibers, leaving a significant gap regarding other abundant, non-food palm residues. This gap includes the pruning waste of ornamental palms widely planted in urban and peri-urban landscaping, whose lignocellulosic composition and particle morphology have not been systematically linked to the flexural response of particulate epoxy composites [1,2,3].

*Washingtonia robusta*, an ornamental palm species extensively cultivated in urban areas of central Chile, generates large volumes of dry leaf stalk residue through routine pruning, most of which is currently disposed of without valorization. Particulate epoxy composites are known to be sensitive to reinforcement granulometry, filler loading, and interfacial quality [2,4,5,7]. Fractographic evidence links these variables to flexural performance through mechanisms such as particle pull-out and crack deflection [3,6,8,9]. A systematic experimental assessment of *Washingtonia robusta* particles as epoxy reinforcement is therefore warranted. The present study addresses this gap by designing, fabricating and characterizing an epoxy particulate composite reinforced with *Washingtonia robusta* residue, examining the influence of reinforcement granulometry on the resulting mechanical and microstructural behavior. The aim is to provide, for the first time, a systematic assessment of the flexural behavior and fracture mechanisms of this locally abundant and currently underutilized lignocellulosic residue, as a first step toward evaluating its potential as a sustainable reinforcement for semi-structural epoxy composite applications.

## 2. Materials and Methods

### 2.1. Materials

The materials used in this study were selected to reproduce, in composite form, the valorization pathway of an urban ornamental palm residue combined with a conventional thermosetting matrix system. Three constituents were used: (i) a particulate vegetal reinforcement derived from *Washingtonia robusta* residue, (ii) a Bisphenol A/F epoxy resin as the polymer matrix, and (iii) a cycloaliphatic amine hardener as the curing agent.

**Reinforcement**. The particulate reinforcement was obtained from the dry leaf stalk residue of *Washingtonia robusta*, collected from pruning operations carried out on specimens growing in the gardens of the Admiral Félix Aguayo Bastidas Marine Infantry Fort, in Concón, Valparaíso Region, Chile. This lignocellulosic byproduct generated routinely as urban green waste was used as received without any chemical surface treatment in order to evaluate its performance as a low-cost unmodified reinforcement.

**Polymer matrix**. The matrix was a commercial Bisphenol A/F epoxy resin (YD-114F, Epokukdo, Kukdo Chemical Co., Ltd., Seoul, Republic of Korea), a low-viscosity liquid diluted with an aliphatic glycidyl ether (C12–C14), selected for its good impregnation capacity, low volatile organic compound content and suitability for solvent free coating and casting systems.

**Curing agent**. Crosslinking of the epoxy matrix was achieved with a cycloaliphatic amine-modified hardener (KH-813, Kukdo Chemical Co., Ltd., Seoul, Republic of Korea), a low-viscosity, solvent-free curing agent suitable for room-temperature cure systems. Its characteristic properties, together with the resin’s, were obtained from the manufacturer’s technical data sheets and are summarized in Table 1 and Table 2.

Together, these three constituents—vegetal particulate reinforcement, epoxy resin and amine hardener—formed the basis for the fabrication of the reference (neat resin) and reinforced composite plates described in the following subsections.

### 2.2. Methods

#### 2.2.1. Reinforcement Preparation

**Figure 1:**
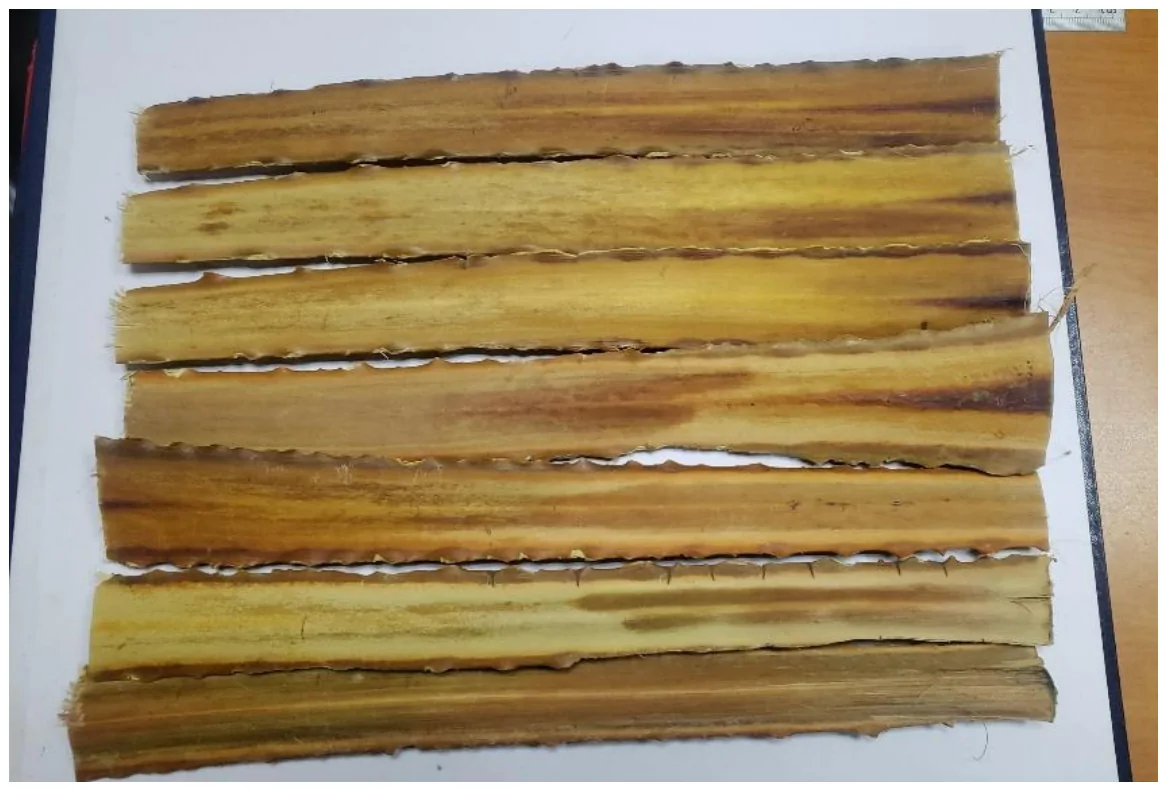
Dry leaf stalk segments of *Washingtonia robusta*, collected as pruning residue prior to conditioning.

**Figure 2:**
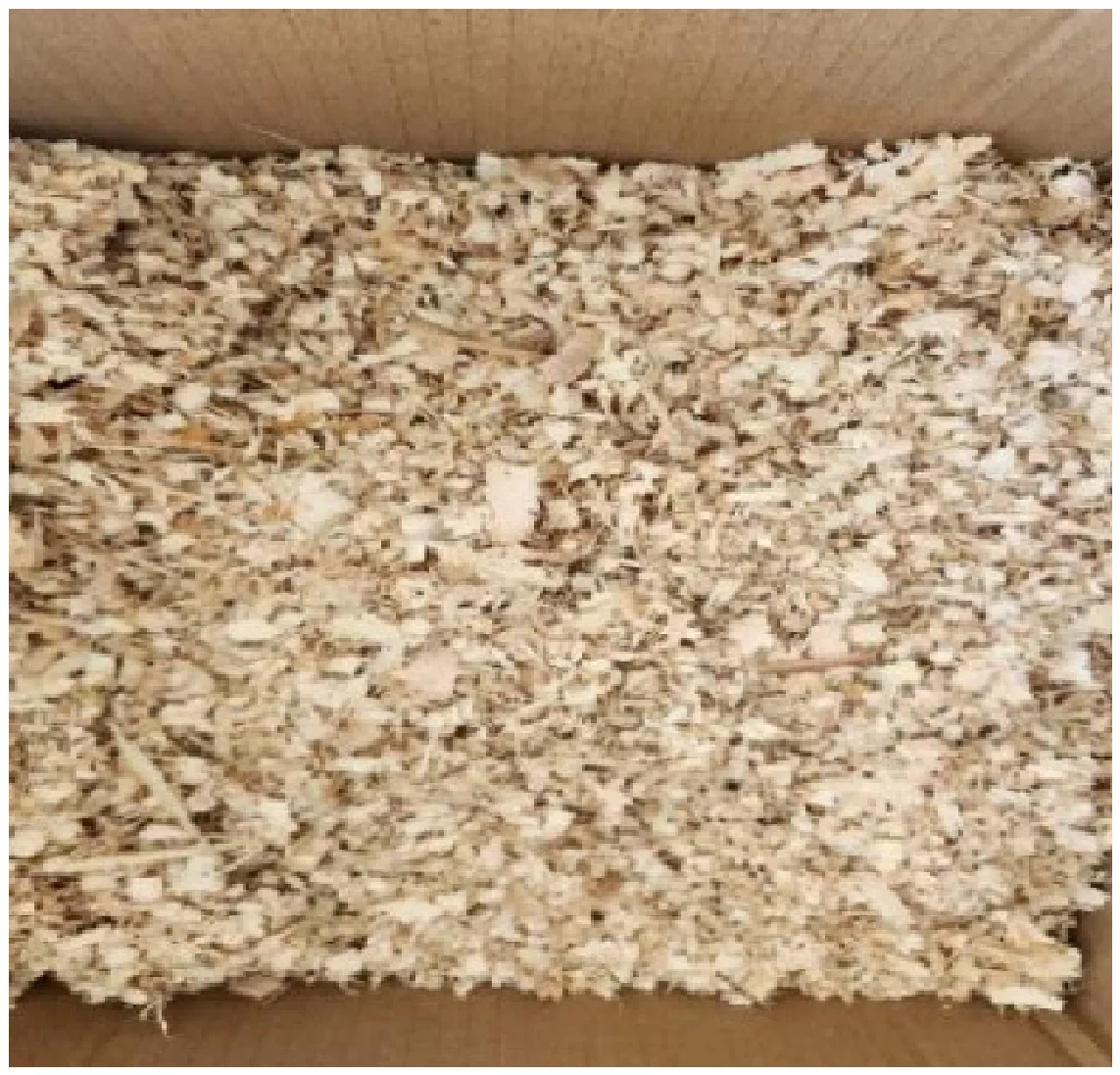
Chipped/brushed fiber material after manual mechanical conditioning, prior to grinding.

The lignocellulosic reinforcement was obtained from dry leaf stalk residue of *Washingtonia robusta* collected from pruning operations in Concón, Valparaíso Region, Chile (Figure 1). The raw material was first conditioned through manual brushing to remove surface impurities, regularize bundle geometry and promote partial defibrillation of the fibrous tissue (Figure 2), thereby increasing effective surface area and exposing hydroxyl groups relevant to subsequent matrix interaction.

Drying was carried out in two complementary stages. The chipped stalks were first air-dried at ambient temperature (~22 °C) over a three-week period, reducing moisture content from approximately 85% to 60%, followed by oven drying at 60 °C prior to composite fabrication to eliminate residual hygroscopic moisture and improve dimensional stability. The final moisture content after oven drying was not directly quantified; ambient temperature and relative humidity during fabrication were monitored using a DHT11 sensor system (Aosong (Guangzhou) Electronics Co., Ltd., Guangzhou, China), which measures environmental conditions rather than the moisture content of the reinforcement material itself.

**Figure 3:**
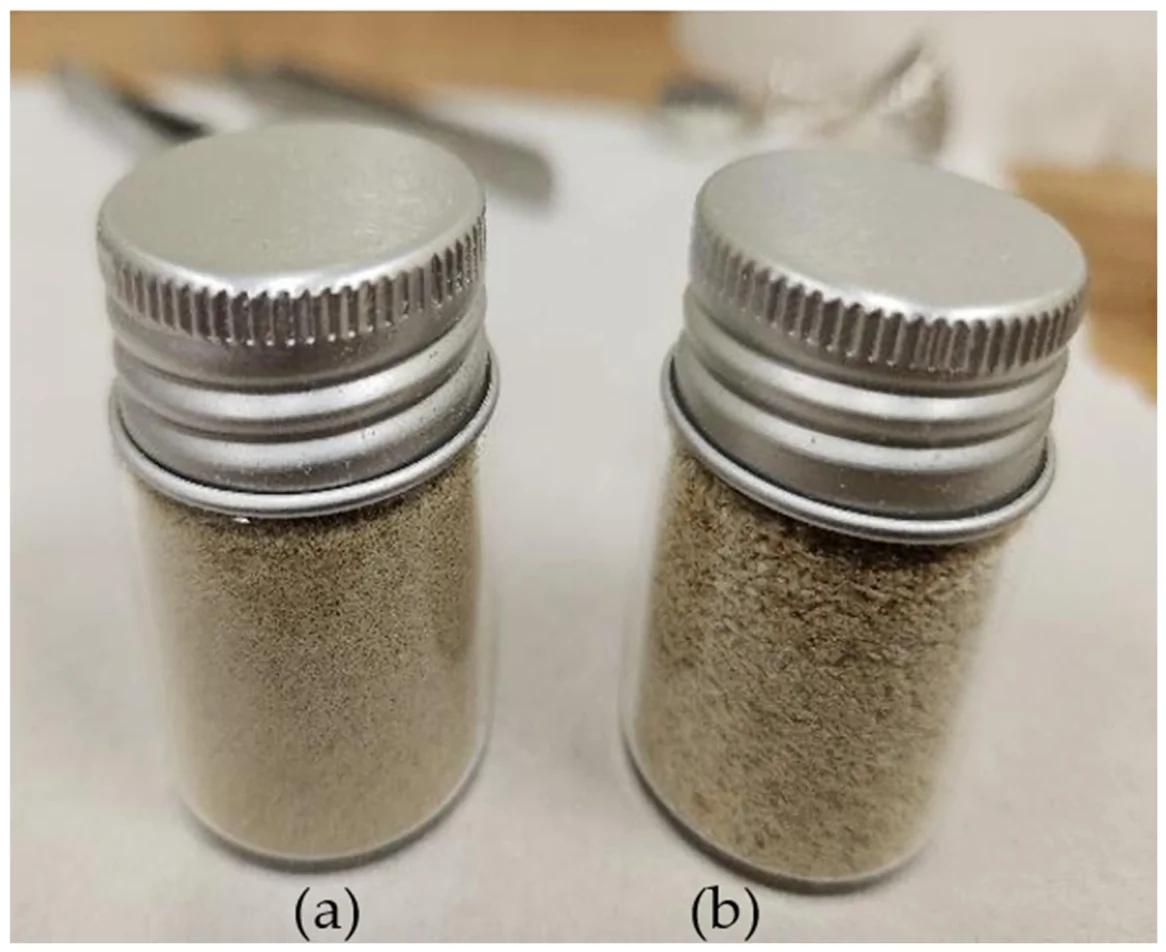
Granulometric fractions of *Washingtonia robusta* reinforcement obtained by sieving: (**a**) fine fraction; (**b**) coarse fraction.

The dried material was then reduced in size using a domestic Thomas-type mill (Los Robles S.A., Santiago, Chile) operating by impact and friction, generating irregular particles with angular and pseudo-spherical morphologies characteristic of brittle lignocellulosic fracture. Milling time was controlled to avoid excessive temperature rise that could compromise the thermal stability of hemicellulose and lignin. The resulting particles were sieved through 1 mm and 200 µm mesh screens to obtain two defined granulometric fractions. Particles passing the 200 µm screen were classified as the fine fraction (<200 µm; Figure 3a). Particles retained between the 200 µm and 1 mm screens were classified as the coarse fraction (200 µm–1 mm; Figure 3b). These fractions were later stored and evaluated individually and in a mixed-fraction condition, obtained by combining the fine and coarse fractions in a 50:50 mass ratio.

The particle size distribution of the reinforcement was determined by sieving, yielding two granulometric fractions, and was subsequently verified by scanning electron microscopy (SEM, Carl Zeiss AG, Oberkochen, Germany). Two magnification levels (40× and 300×) on three different samples of both the fine and coarse material were used, from which *n* individual particle measurements were obtained. Table 3 presents the average particle size obtained for the fine and coarse fractions, along with the number of measurements *n* performed for each. These fractions were subsequently used individually (RF, RG) and in a blended particle size mixture (RM).

**Figure 4:**
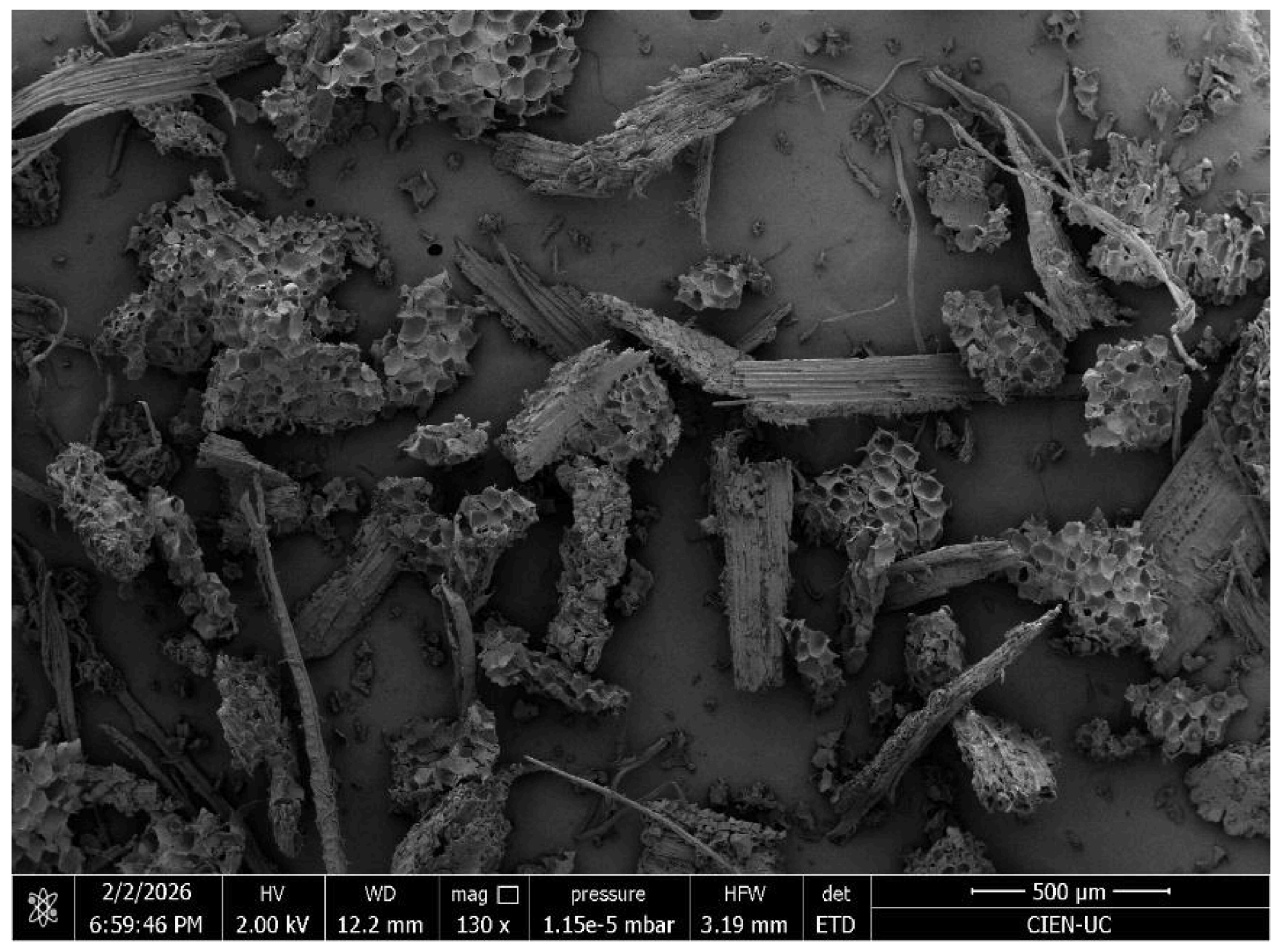
SEM micrograph (130×) of sieved particles.

**Figure 5:**
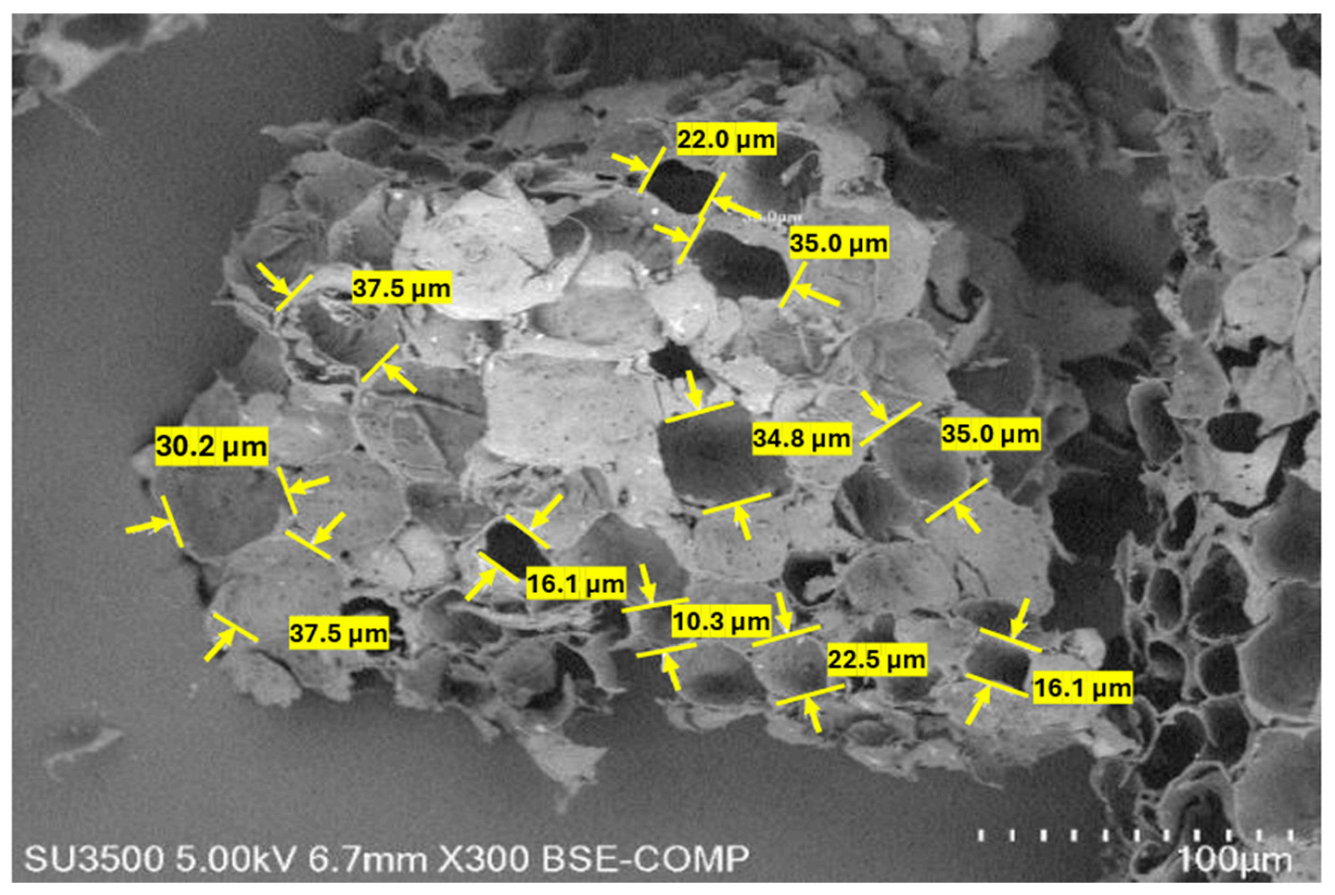
SEM micrograph (300×) with lumen diameter measurements.

SEM analysis (Figure 4) revealed a heterogeneous particle morphology composed of three types of fragments. Elongated, fibrous particles are remnants of the stalk’s vascular structure. Angular fragments result from the brittle fracture behavior characteristic of the lignocellulosic material during milling. Pseudo-spherical agglomerates with a honeycomb-like cellular structure correspond to cross-sections of the tissue with preserved lumens. Quantification of these internal cavities (Figure 5) confirmed the presence of cellular lumens of variable diameter, constituting an additional morphological parameter independent of the overall granulometry. The coexistence of elongated, angular and porous particles within a single fraction implies that specimens sharing the same particle size may still differ in their internal geometry and available bonding surface, factors that could act as an additional source of mechanical variability beyond granulometry alone.

#### 2.2.2. Composite Fabrication

The epoxy resin was preheated in a water bath to approximately 40 °C and catalyzed at the manufacturer-recommended level (50 phr), following the technical specifications of the resin–hardener system. The sieved reinforcement particles were incorporated progressively under constant manual agitation to promote homogeneous wetting and to break up micro-agglomerates, targeting a nominal reinforcement content of 20% by volume for all reinforced formulations. Environmental conditions (temperature and relative humidity) were continuously monitored during mixing, drying, and curing using an Arduino-based acquisition system (Arduino S.r.l., Monza, Italy) with a DHT11 sensor. To keep process variables within controlled ranges, recorded conditions remained within 45–60% relative humidity and approximately 25 °C throughout fabrication for all specimen families (RS, RF, RG, RM). After mixing, the system was allowed to rest briefly to release entrapped air and stabilize viscosity before casting.

The mixture was cast into release agent-treated molds and subjected to progressive compression up to 60 bar, promoting resin infiltration into the reinforcement interstices, reducing internal void content and improving plate geometric uniformity.

An initial cure was carried out at room temperature for 24 h, followed by a two-stage post-cure treatment (2 h at 60 °C and 2 h at 80 °C) to complete crosslinking of the epoxy network.

Four specimen families were manufactured: neat resin (RS, reference), reinforced composites with fine (RF), coarse (RG) and mixed-fraction (RM) particles, with five specimens per condition. After demolding, plates were machined using a manual hacksaw to obtain flexural test specimens with nominal dimensions of 210 mm (length) × 18 mm (width) × 9 mm (thickness). Dimensional verification was performed using a caliper to ensure compliance with the geometric tolerances required for testing.

#### 2.2.3. Experimental Analysis Methods

Flexural behavior was evaluated by three-point bending in accordance with ISO 178:2019 [10], using a universal testing machine (WDW-200E, Jinan Kason Testing Equipment Co., Ltd., Jinan, China ) equipped with a digital data acquisition system for continuous force–deflection recording. Specimens were tested with a support span of approximately 154 mm (span to thickness ratio ≈ 17) and a crosshead speed of 2 mm/min, following an initial preload to ensure proper seating on the supports. Neat resin specimens were tested under an extended acquisition window (up to 1800 s) to capture their viscoelastic response, while reinforced specimens were tested to fracture. Due to this difference in acquisition protocol, the RS dataset is treated in this study as a qualitative observation of the matrix’s large-deformation viscoelastic response, distinct from the standard ISO 178:2019 [10] characterization applied to the reinforced systems (RF, RG, RM). Deflection, stress, and modulus values are therefore not directly compared between RS and the reinforced families. Flexural stress and flexural modulus were calculated according to Equations (1) and (2), respectively.

(1) $$\sigma =\frac{3PL}{2bh^{2}}$$

where *P* is the applied load, *L* is the support span, *b* is the specimen width and *h* is the specimen thickness.

(2) $$E_{f}^{secant}=\frac{L^{3}m}{4bh^{3}}$$

where *m* is the slope of the initial linear region of the force–deflection curve, determined individually for each specimen within the deflection range corresponding to the 0.05–0.25% flexural strain interval defined by ISO 178:2019 [10].

Fracture surfaces were examined by scanning electron microscopy (SEM) using a Hitachi SU3500 microscope (Hitachi High-Tech Corporation, Tokyo, Japan) equipped with a Bruker XFlash 410M energy-dispersive X-ray (EDX) detector (Bruker Nano GmbH, Berlin, Germany) (133 eV resolution at Mn Kα). Imaging combined secondary electron (SE) detection, emphasizing surface topography and fracture morphology, with backscattered electron (BSE) detection, sensitive to compositional/density contrast. Given the insulating nature of the epoxy biocomposites, low accelerating voltages and a conductive coating were used to minimize charging artifacts. EDX analysis complemented the morphological observations by providing elemental composition data (e.g., Si, K, Mg, Fe), allowing for confirmation of organic/inorganic residues and compositional differences across the reinforcement and matrix phases. Fractographic features including particle pull-out, matrix–particle debonding and crack deflection were correlated with the mechanical response of each specimen family to relate microstructural damage mechanisms to the observed flexural performance.

## 3. Results

### 3.1. Fractographic Analysis (SEM)

#### 3.1.1. Fractographic Analysis—RS Family (Neat Resin)

SEM observations reported in this section are based on a limited number of representative micrographs per specimen family (one to two fields of view at each magnification) and are intended as qualitative fractographic interpretations rather than statistically quantified measurements. Comparative descriptors such as “larger”, “more numerous”, or “relatively strong interfacial adhesion” refer to visually apparent differences between the observed fields and should be read as indicative trends supporting the mechanical results, not as quantified metrics.

**Figure 6:**
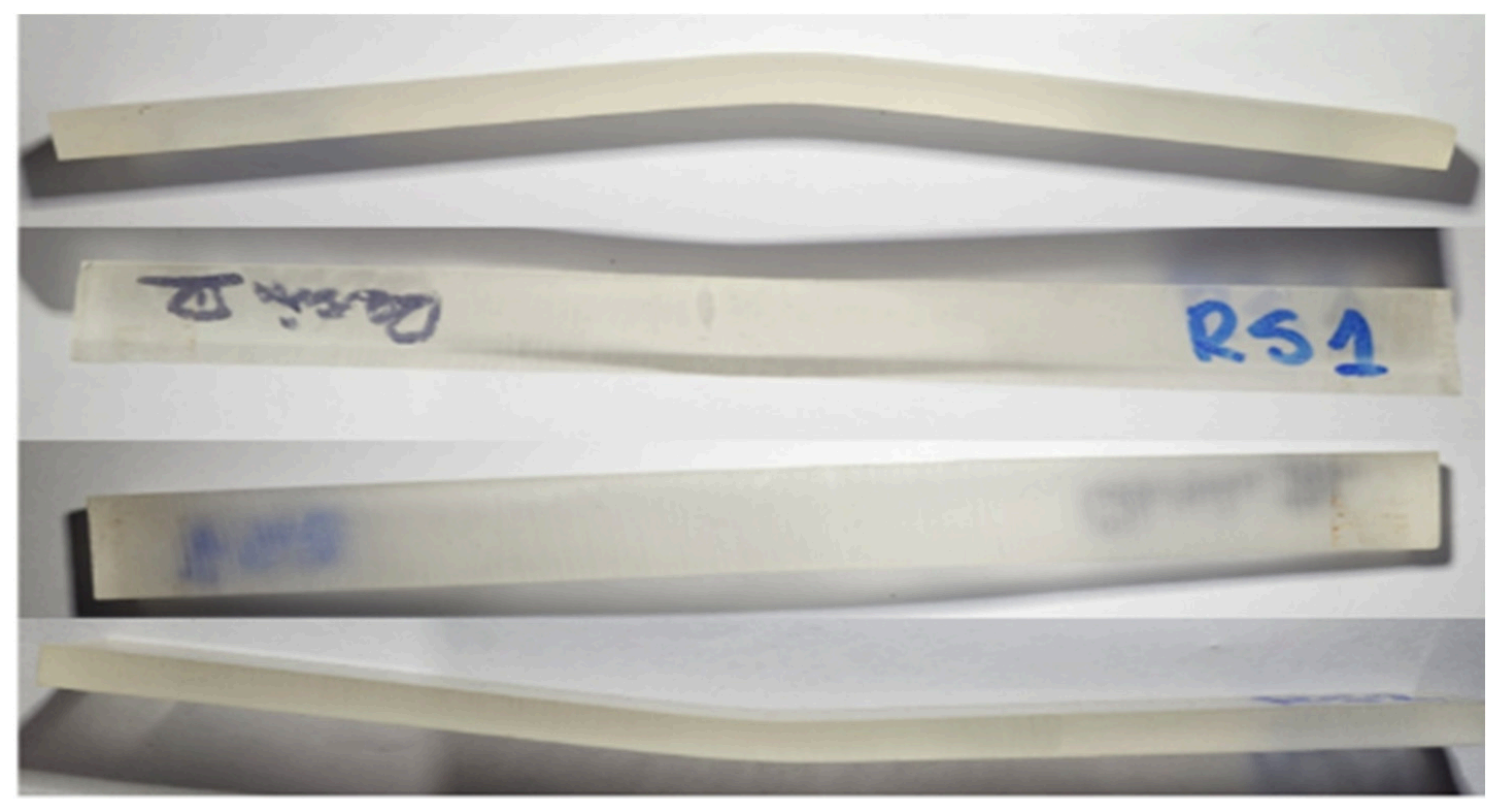
RS specimens after bending (unfractured).

Figure 6 shows the macroscopic photographic record of the neat resin (RS) specimens after the three-point bending test, evidencing permanent curvature without separation of the material. This result indicates that the RS specimens did not reach fracture during the test, but rather underwent sustained bending deformation. This observation is consistent with the absence of abrupt stress drops in the stress–deflection curves.

**Figure 7:**
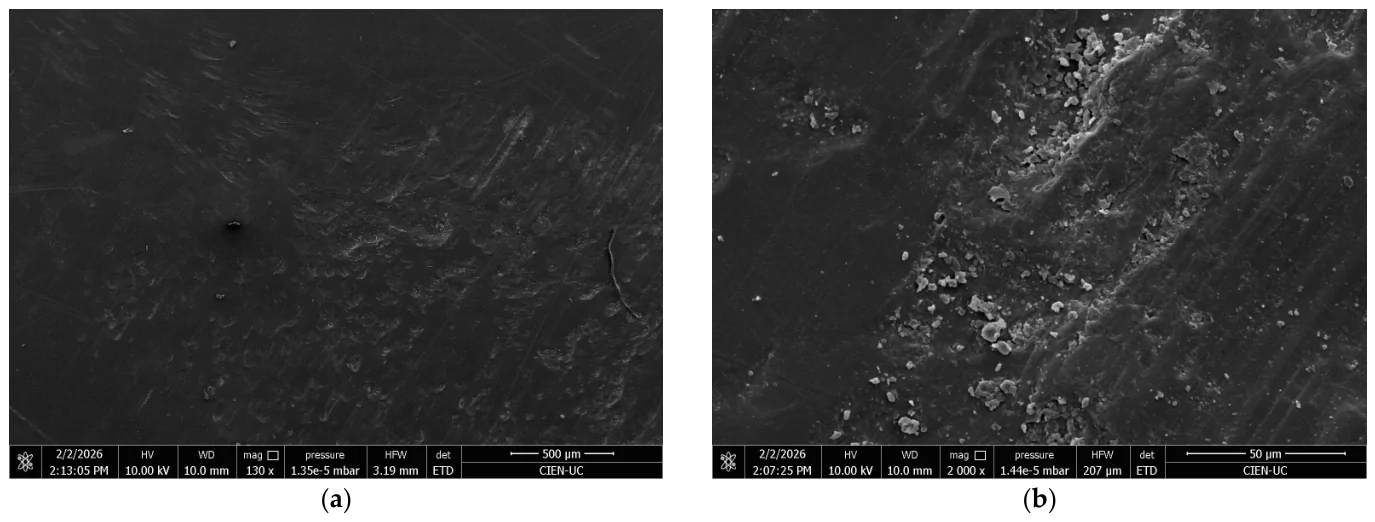
SEM micrograph of RS1 specimen surface: (**a**) 130× and (**b**) 2000×.

Figure 7 corresponds to the surface of specimen RS1 in the contact zone with the loading punch. Panel (a) shows the surface at 130× (HFW 3.19 mm, 500 µm scale bar), and panel (b) at 2000× (50 µm scale bar), using a secondary electron detector (ETD, integrated component of the Hitachi SU3500, Hitachi High-Tech Corporation, Tokyo, Japan) at 10 kV. It is important to note that these micrographs do not correspond to a fracture surface: as shown by the photographic record in Figure 4, specimen RS1 did not fracture during the test, but instead accommodated the applied load through sustained deformation.

At low magnification (Figure 7a), the surface is predominantly smooth and homogeneous. A subtle pattern of diagonally oriented wavy marks is consistent with surface traces from the contact and displacement of the punch during sustained bending. A fine line toward the right edge of the image is consistent with an incipient microcrack that did not propagate far enough to produce separation of the material. This behavior-localized microstructural damage without catastrophic fracture is consistent with a response dominated by viscoelastic relaxation of the epoxy matrix under sustained loading, in which deformation is progressively accommodated rather than concentrated at a single crack front [11,12,13].

At higher magnification (Figure 7b), small light-toned granular aggregates are identified, heterogeneously distributed across the surface, along with a fine striated background texture. These aggregates are consistent with local defects associated with the curing process: zones of incomplete mixing between resin and hardener, residual microbubbles, or small cross-linking heterogeneities. Such defects may act as local stress concentrators without triggering macroscopic fracture, in line with what would be expected for cured surfaces of neat epoxy resins [14,15]. No relevant energy dissipation mechanisms—such as structural bridging or extensive pull-out zones—are observed at this scale. This is consistent with the absence of particulate reinforcement capable of deflecting damage propagation.

#### 3.1.2. Fractographic Analysis—RF Family (Fine Fraction)

**Figure 8:**
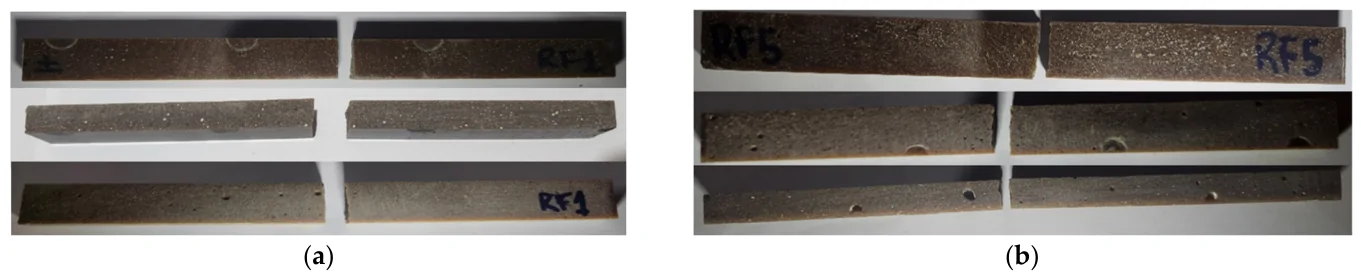
RF specimens after three-point bending fracture: (**a**) RF1 and (**b**) RF5.

The macroscopic photographs (Figure 8a,b, corresponding to RF1 and RF5) show the upper surface of the specimens. A heterogeneous distribution of light-colored particles is dispersed within the epoxy matrix, along with small pinpoint pores visible as dark marks along the longitudinal edge. What is most relevant, however, is the side view of both fractured segments: in all three specimens, the fracture line does not follow a single plane perpendicular to the loading axis, but rather an irregular, “sawtooth-like” path running along the edge of the specimen. This morphology constitutes direct macroscopic evidence of a crack front that repeatedly changes direction during propagation, in contrast to the predominantly flat and rectilinear fracture observed in the neat resin (RS). This type of irregular trajectory is characteristic of particulate composites in which the reinforcement acts as a physical obstacle that deflects the crack away from its lowest-energy plane, increasing the total fracture area and, therefore, the energy required for damage propagation [13,16].

**Figure 9:**
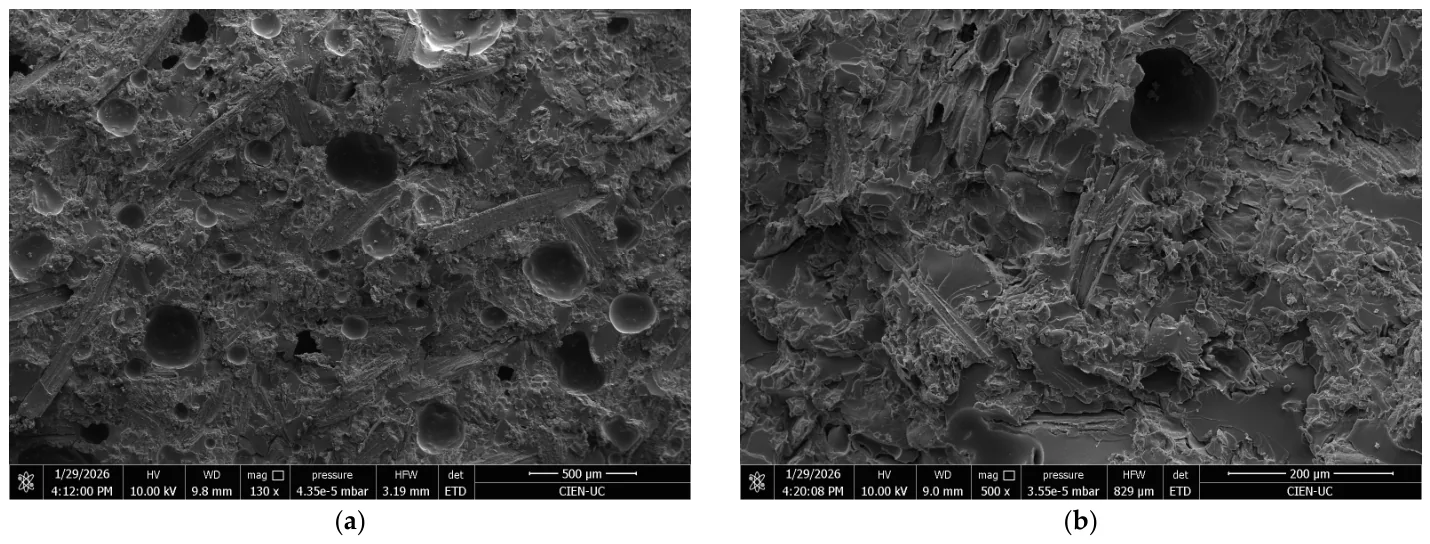
SEM micrographs of the RF5 specimen fracture surface: (**a**) 130× and (**b**) 500×.

Figure 9 shows the SEM micrographs corresponding to the RF specimens. At the microstructural level, the RF5 micrograph (130×; Figure 9a) shows a topography that is notably rougher and more heterogeneous than that observed in RS at the same scale. Multiple stepped fracture planes and local changes in surface orientation are consistent with a non-coplanar crack front. At higher magnification (RF5, 500×) (see Figure 9b), zones of interaction between the epoxy matrix and the reinforcing particles can be distinguished more clearly, including possible cavities associated with particle detachment or extraction (pull-out) and regions of local debonding at the matrix–reinforcement interface. This combination of features is characteristic of particulate systems in which fine-sized reinforcement is well dispersed within the matrix. It favors a distributed interaction between crack and reinforcement, rather than propagation concentrated along a single front [14,17].

Unlike RS, which did not fracture, RF specimens do reach fracture, but within a strain range (~10–14.5 mm) and with a somewhat less pronounced softening slope. The deflection and multiplication of fracture planes, observed both macroscopically and microscopically, explains this greater damage tolerance, at least in part. Energy that would otherwise concentrate at a single crack front is partially dissipated through the generation of additional fracture surface and through local interaction with the particles [13,16].

#### 3.1.3. Fractographic Analysis—RG Family (Coarse Fraction)

**Figure 10:**
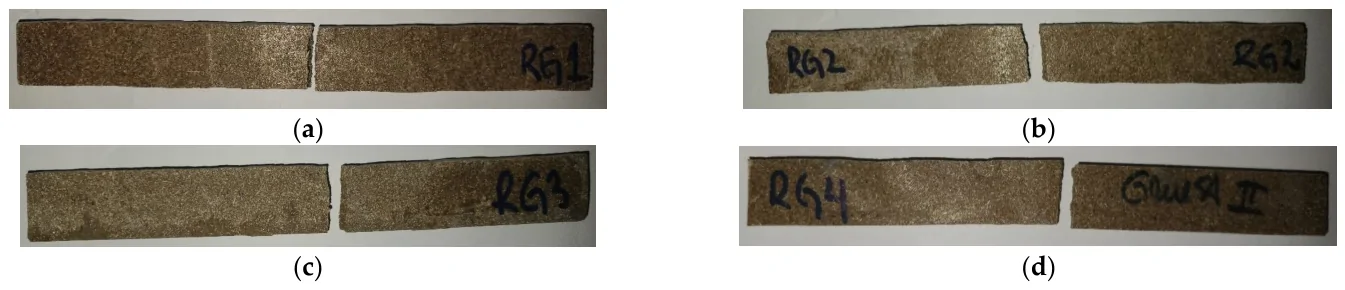
RG specimens after three-point bending fracture: (**a**) RG1; (**b**) RG2; (**c**) RG3; (**d**) RG4.

Figure 10 shows the fractured RG specimens after three-point bending testing (RG1–RG4). Unlike the RF family, where the fracture line followed an irregular, sawtooth-like path along the specimen edge, the RG specimens exhibit a comparatively more rectilinear fracture line across the four samples, with the two broken segments aligning along a single, relatively direct plane. This more planar fracture geometry suggests a crack front that propagated with fewer directional changes than in RF. This interpretation is consistent with a failure mode less dominated by particle-induced deflection and more governed by localized stress concentration [16,18].

**Figure 11:**
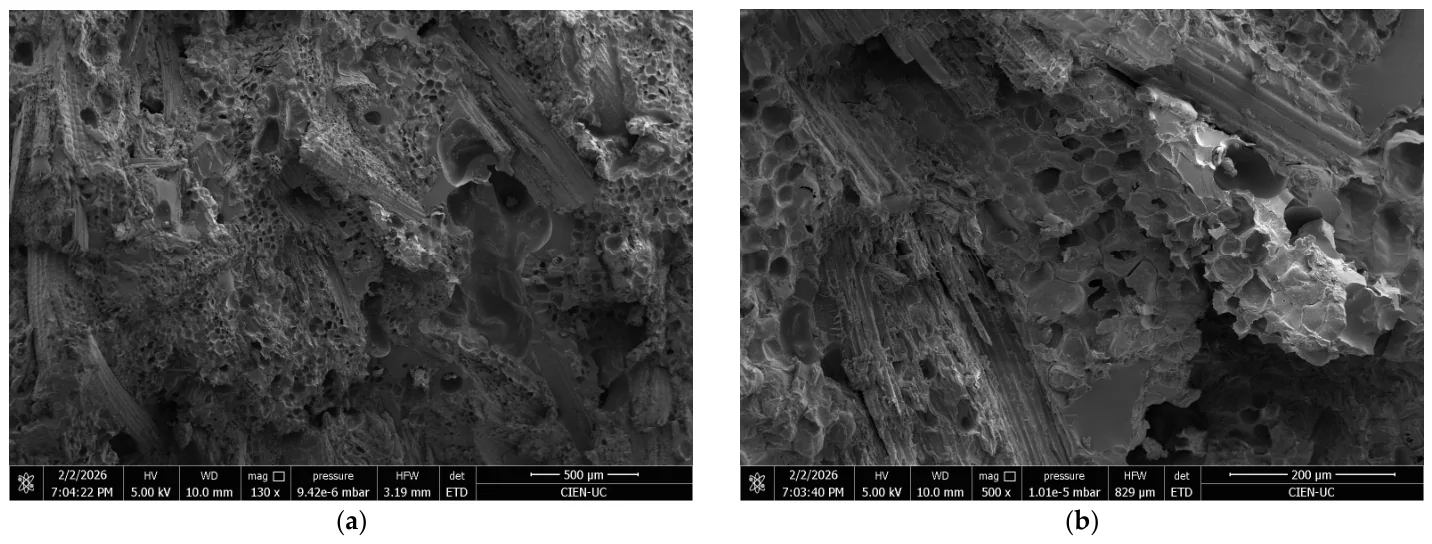
SEM micrographs of the RG fracture surface: (**a**) 130× and (**b**) 500×.

Figure 11 shows the SEM micrographs of the RG fracture surface at 130× (a) and 500× (b). At both magnifications, the surface exhibits numerous cavities and elongated voids distributed across the fibrous matrix texture, several of which appear notably larger than those observed on the RF fracture surfaces at comparable scales. These larger cavities are consistent with the pull-out or detachment of coarse *Washingtonia robusta* particles from the epoxy matrix. This mechanism is widely associated with weak particle–matrix interfacial bonding in natural particle-reinforced composites, where inadequate wettability and limited chemical/physical adhesion leave behind open voids upon particle extraction [18,19]. The presence of numerous large voids on the fracture surface is a recognized indicator of premature interfacial failure, since these cavities act as stress concentrators that reduce the effective load-bearing cross-section and accelerate crack coalescence [14,16,19].

This fractographic evidence is consistent with the mechanical behavior of the RG family, which exhibited the narrowest strain-to-fracture range among the three reinforced families (~8–11 mm) and the steepest softening slope. Together, the relatively planar crack path and the larger, more numerous pull-out cavities observed in the SEM images point to the same underlying cause. The coarse particle size itself acts as a critical flaw within the matrix, lowering the material’s overall damage tolerance compared to RF [16,18].

#### 3.1.4. Fractographic Analysis—RM Family (Mixed Fraction)

**Figure 12:**
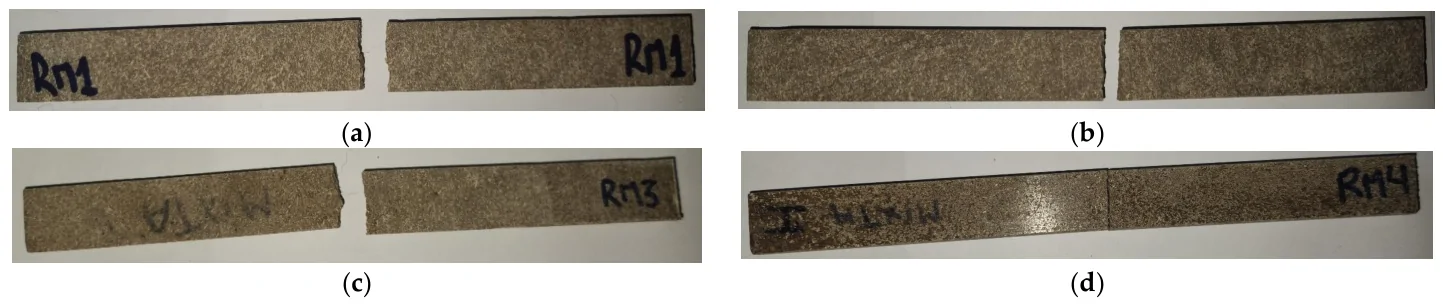
RM specimens after three-point bending fracture: (**a**) RM1; (**b**) RM2; (**c**) RM3; (**d**) RM4.

Figure 12 shows the fractured RM specimens after three-point bending testing: (a) RM1, (b) RM2, (c) RM3 and (d) RM4. The RM specimens display a more variable fracture pattern than RG (single, relatively planar line) or RF (pronounced sawtooth path). RM1 (a) and RM3 (c) show a moderately oblique fracture line. RM4 (d) exhibits a longer, more diagonal separation extending across a greater portion of the specimen edge. In RM2 (b), the two segments remain almost seamlessly aligned, with the fracture line barely distinguishable. This variability, together with the generally limited and localized nature of the visible separation, is consistent with a failure process in which crack extension was repeatedly interrupted or redirected, rather than propagating freely through the cross-section. This pattern is associated with effective energy dissipation in hybrid particulate systems [13,17].

**Figure 13:**
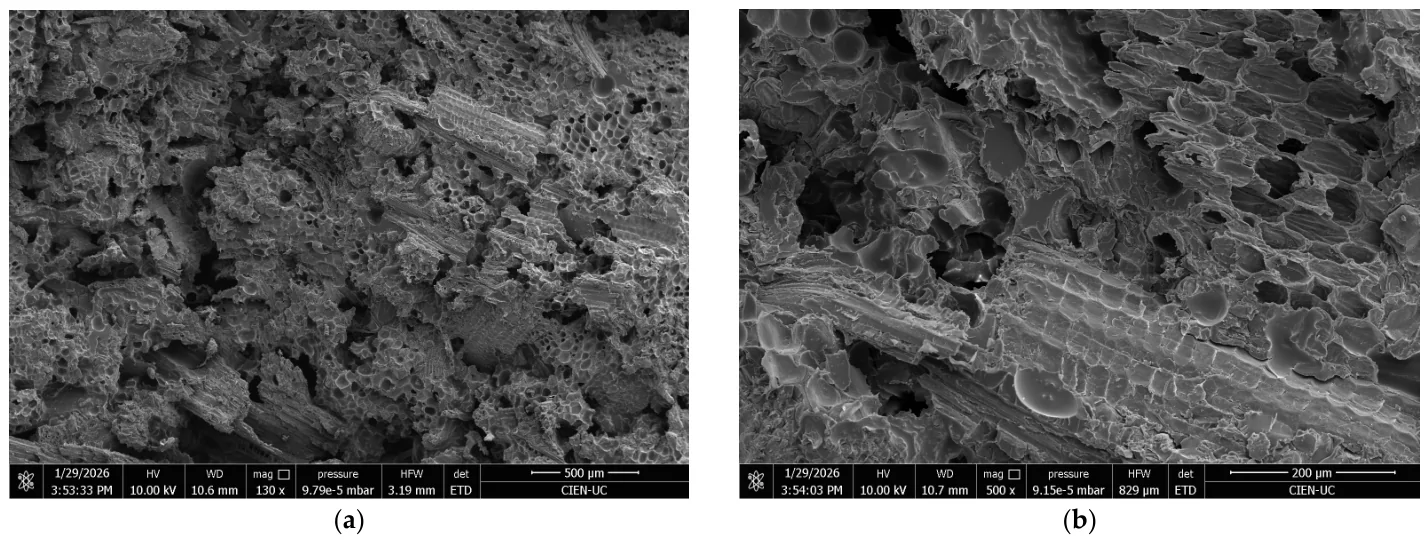
SEM micrographs of the RM fracture surface: (**a**) 130× and (**b**) 500×.

Figure 13 shows the SEM micrographs of the RM fracture surface at 130× (a) and 500× (b). Both images reveal a markedly cellular, honeycomb-like texture superimposed on the fibrous matrix, corresponding to the exposed vascular bundle structure of the *Washingtonia robusta* particles rather than a clean, flat interfacial separation. At lower magnification (a), this cellular texture is visible across a broad area of the surface alongside dispersed fibrous strands. At higher magnification (b), individual cell lumina are clearly visible within the particle’s internal fibrous structure, with epoxy matrix material still adhering to the cell walls. This morphology indicates that the crack propagated, at least partially, through the internal cellular structure of the reinforcing particles, rather than exclusively along the particle–matrix interface. This is generally interpreted as suggesting improved interfacial stress transfer: when bonding is weak, cracks tend to deflect around intact particles and produce clean pull-out cavities, whereas fracture through the particle itself is consistent with more effective stress transfer into the reinforcement before failure [14,18,20].

### 3.2. Three-Point Bending Test

This section presents the mechanical results obtained from three-point bending tests on four systems: neat epoxy resin (RS), used as the reference material and three formulations reinforced with *Washingtonia robusta* particles of different particle size—fine (RF), coarse (RG) and mixed (RM).

**Figure 14:**
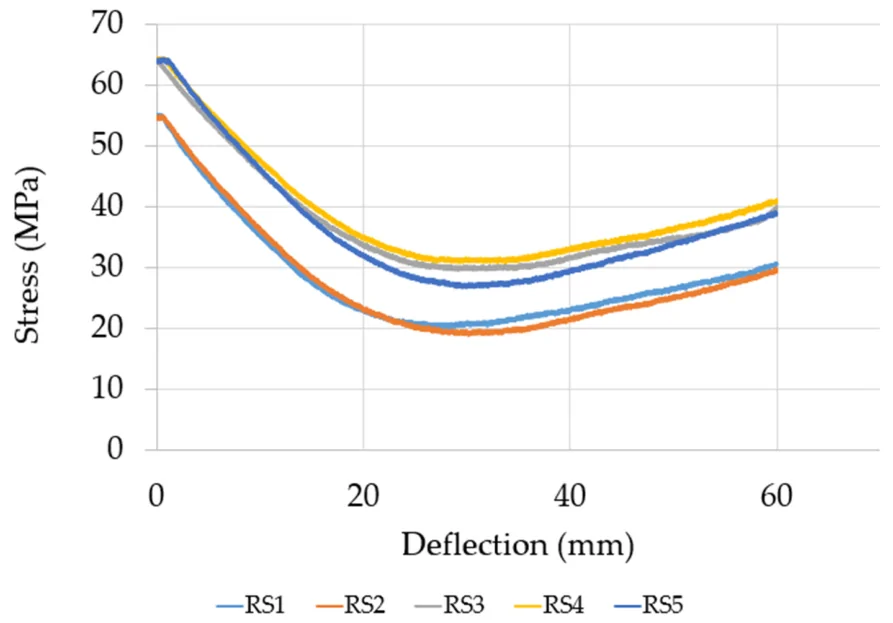
RS flexural stress–deflection response.

The stress–deflection curve for the neat resin specimens (RS1–RS5) shows a characteristic non-monotonic trend, with three clearly distinguishable regimes (see Figure 14). In the initial stage (0–25 mm), stress decreases continuously from high initial values (55–64 MPa) down to a minimum of approximately 20–32 MPa. This behavior is consistent with the viscoelastic relaxation phenomenon reported for epoxy resins under sustained flexural loading, where reorganization of the polymer network and internal stress redistribution produce progressive stress decay without loss of structural continuity [11,21,22,23]. The consistency of this trend across the five specimens, with a relatively narrow spread between curves, supports the repeatability of the manufacturing process and of the matrix’s intrinsic behavior. This agrees with observations by [24] for epoxy systems tested under three-point bending, where the mechanical response proved reproducible among specimens of the same series. This absence of fracture is corroborated at the microstructural level. The SEM micrographs of specimen RS1 (Figure 6 and Figure 7) show a predominantly smooth, homogeneous surface, with only an incipient microcrack and localized curing-related defects. There is no evidence of a propagated fracture front. This observation is consistent with a matrix that accommodated the applied deformation through viscoelastic relaxation rather than crack growth [11,12].

Between 25 and 30 mm of deflection, the curves reach a relatively stabilized stretch, with minimum stresses ranging from 20 MPa (RS1, RS2) to 32 MPa (RS4, RS5). This plateau can be interpreted as a transient quasi-equilibrium viscoelastic condition, in which the rate of stress relaxation decreases significantly and the response becomes less sensitive to further increases in deflection [12,22]. This type of stabilization has been documented in neat epoxy resins and their composites, where the system tends toward a state of sustained deflection without evolving into catastrophic damage [21].

From approximately 30 mm onward, all curves show a gradual increase in stress until the end of the recorded data (60 mm), with no evidence of fracture in any specimen. This apparent hardening should not be interpreted as a recovery of the material’s intrinsic stiffness. It instead reflects geometric nonlinearity inherent to large deflections in the three-point bending test. As deformation approaches an appreciable fraction of the support span, the small-deflection assumptions underlying the conventional stress calculation cease to hold strictly, producing a progressive deviation from the expected linear behavior. This phenomenon is consistent with nonlinear bilinear viscoelastic-softening stress–strain models recently used to capture the response of epoxy composites subjected to large deformations without immediate structural collapse [13]. ISO 178:2019 [10] limits the conventional calculation of flexural properties to a reference deformation considerably smaller than that reached in this test. The values recorded in the final stretch should therefore be interpreted qualitatively and comparatively, rather than as a strict determination of flexural modulus or strength.

**Figure 15:**
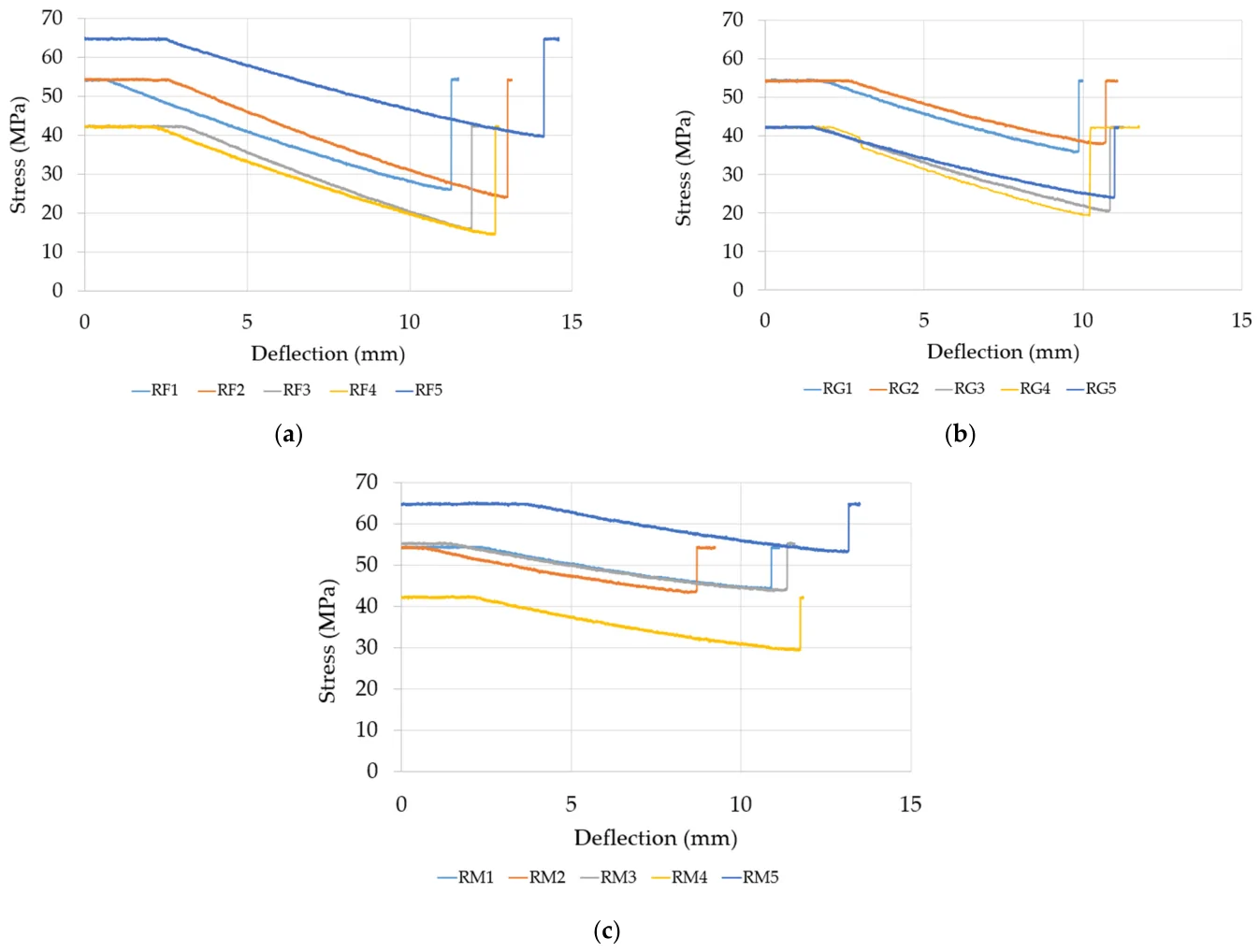
Flexural stress–deflection curves of composites: (**a**) RF (fine fraction), (**b**) RG (coarse fraction) and (**c**) RM (mixed fraction).

In contrast to the large-deflection viscoelastic observation recorded for RS under extended testing, the three reinforced systems (RF, RG, RM) were tested to fracture following the standard ISO 178:2019 [10] procedure. They fractured within a deflection range of approximately 8–14.5 mm, as shown in Figure 15.

The RF specimens (Figure 15a) exhibit initial stresses between 42 and 65 MPa, followed by a continuous decrease to values between 15 and 40 MPa before fracture, identifiable by the characteristic abrupt stress jump at the end of each curve. This comparatively gradual softening, less steep than that observed for RG, is consistent with the crack deflection mechanism identified in the fractographic analysis of RF. There, the fracture path deviates repeatedly rather than propagating along a single plane, increasing the fracture area and the energy required for crack advance [16,18].

The RG specimens (Figure 15b) display the steepest softening slope and trend toward the narrowest deflection-to-fracture range (~8–11 mm) among the reinforced systems, with minimum stresses as low as 18–24 MPa immediately before fracture. This earlier and more abrupt failure is consistent with the larger interfacial defects identified in the fractographic analysis of RG. Coarse particle pull-out leaves behind sizable cavities that act as stress concentrators and accelerate crack coalescence, limiting the material’s capacity to resist crack advance relative to RF and RM [16,18].

The RM specimens (Figure 15c) show the most favorable mechanical response among the reinforced systems, with a gentler stress decline and a trend toward the widest deflection-to-fracture range of the three families. RM1 and RM5 in particular retain stresses above 50 MPa throughout most of the tests. This behavior is consistent with the complementary reinforcement mechanisms identified in the fractographic analysis of RM. Fine particles support matrix continuity, while coarser particles offer additional resistance to crack propagation, together producing the most favorable damage tolerance profile among the four systems evaluated [17,20].

**Figure 16:**
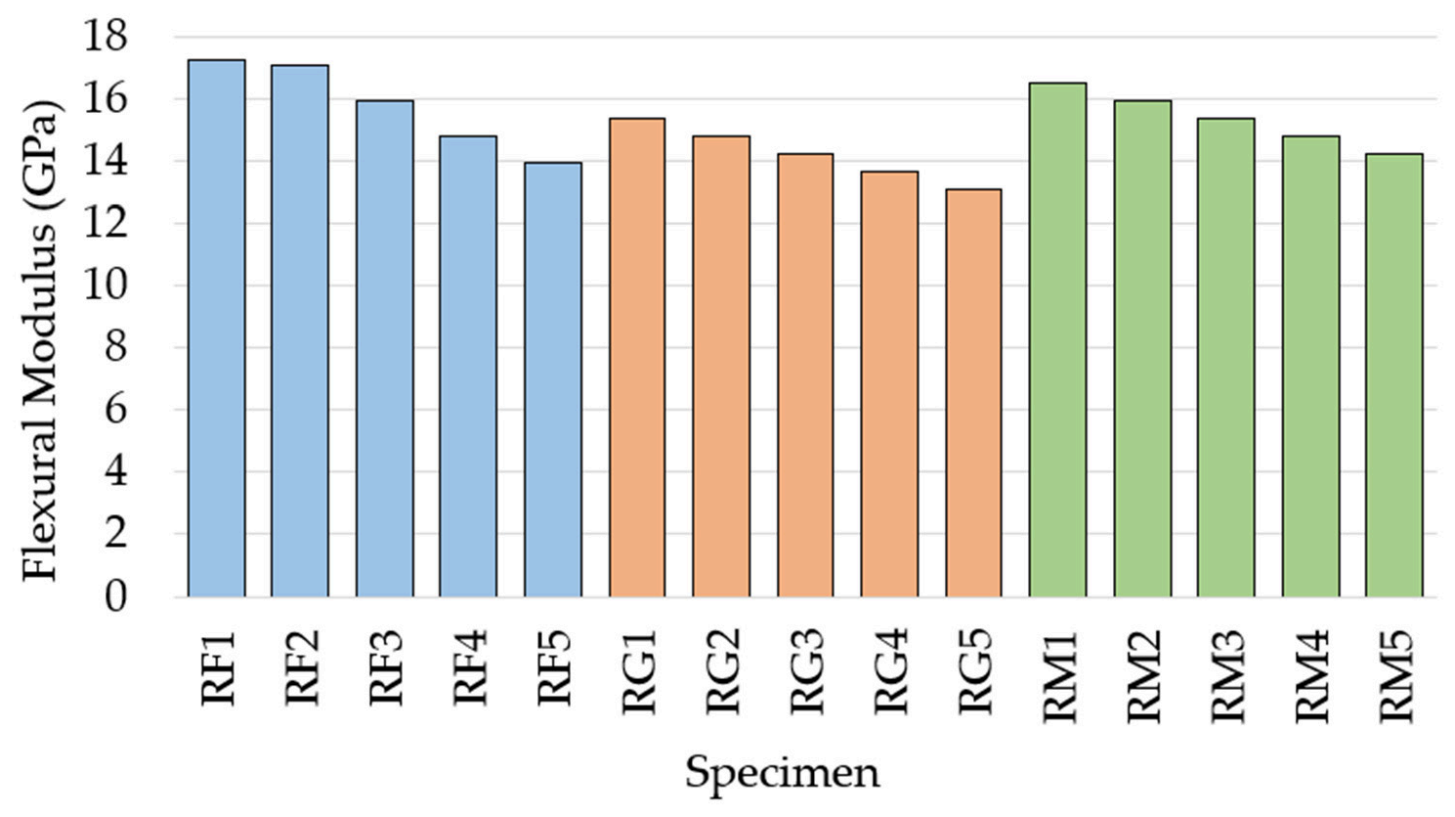
Flexural modulus of the reinforced composites (RF, RG, RM) by specimen.

The flexural modulus obtained for each specimen of the reinforced families (RF, RG, RM) is presented in Figure 16. RF specimens show the highest values overall, ranging from approximately 13.9 to 17.3 GPa (mean ± SD = 15.80 ± 1.43 GPa; CV = 9.1%). RM follows closely, ranging from 14.2 to 16.6 GPa (mean ± SD = 15.36 ± 0.90 GPa; CV = 5.9%). RG consistently shows the lowest values, ranging from 13.1 to 15.4 GPa (mean ± SD = 14.22 ± 0.90 GPa; CV = 6.3%). This ranking is consistent across all five specimens within each family. This consistency suggests a repeatable trend in the effect of particle granulometry on stiffness. However, given the limited number of specimens per family (*n* = 5), these differences are reported as descriptive observations rather than statistically confirmed effects (see Table 4).

Despite RF showing the highest average stiffness, it also displays the greatest relative dispersion among specimens, whereas RG and RM show comparatively more consistent behavior. The neat resin (RS) family is not included in Figure 16, as none of its five specimens developed a linear elastic region meeting the ISO 178:2019 [10] criteria required to compute the secant modulus (Equation (2)); RS instead exhibited a stable load plateau without a defined maximum. This is consistent with the bending-without-fracture behavior discussed before.

To assess whether the observed differences in flexural performance between the reinforced families were statistically robust, a one-way analysis of variance (ANOVA) was performed for each mechanical property. Specimen family (RF, RG, RM) served as the single factor, with individual specimen values (*n* = 5 per family) as replicates. Table 4 summarizes, for each property, the mean and standard deviation (SD) per family, together with the F-ratio and associated *p*-value from the ANOVA, which test whether the mean differences between families are larger than expected from within-family variability alone. Flexural strength showed no significant difference between families (F(2,12) = 0.97, *p* = 0.41). Flexural modulus followed the expected ranking (RF > RM > RG) but did not reach statistical significance at the conventional α = 0.05 threshold (F(2,12) = 2.70, *p* = 0.11). Deflection at fracture showed the strongest trend among the three properties, approaching but not reaching significance (F(2,12) = 3.16, *p* = 0.08). Taken together, these results indicate that the differences in flexural performance among RF, RG, and RM should be interpreted as descriptive trends rather than statistically confirmed effects, given the limited sample size (*n* = 5 per family). The direction of these trends is, nonetheless, consistent with the fractographic evidence discussed.

The higher modulus observed for RF is consistent with the greater specific surface area available for stress transfer in fine-particle systems. Smaller particles provide a larger particle–matrix contact area per unit volume, favoring more efficient load transfer from the epoxy matrix to the rigid reinforcement. This results in a stiffer composite, provided the particles remain well dispersed [16,25]. Milling produced irregular particles with heterogeneous morphology, and the fine and coarse fractions may also differ in shape, aspect ratio, and packing behavior. These factors may contribute alongside particle size to the mechanical differences reported here. Conversely, the comparatively lower modulus of RG is consistent with the fractographic evidence obtained for this family (Figure 10 and Figure 11), where large interfacial cavities and weak particle–matrix bonding were observed. A reduced effective load-bearing cross-section and inefficient stress transfer at the coarse particle–matrix interface may plausibly contribute to this depressed elastic response [18,19]. Possible differences in particle packing, resin-rich regions, or interfacial defects suggested by the fracture surfaces may also contribute, rather than particle size alone.

RM shows an intermediate-to-high modulus, close to that of RF despite containing a substantial fraction of coarse particles. This behavior suggests that, in the mixed-granulometry system, the fine fraction contributes disproportionately to stiffness by providing efficient, well-distributed stress transfer. The coarse fraction, despite its lower individual contribution to modulus, is qualitatively associated with the superior damage tolerance and crack deflection behavior already identified for RM in the fractographic and flexural deflection-to-fracture analysis. This proposed synergistic mechanism is based on fractographic observation and mechanical deflection ranges rather than on quantified crack tortuosity, pull-out, or fracture energy metrics and should be regarded as a qualitative interpretation rather than a confirmed mechanistic explanation. Within this scope, RM offers the most favorable mechanical profile among the three reinforced systems, combining a flexural modulus comparable to that of RF with the damage tolerance trends associated with its hybrid granulometry [15,16].

When compared with reported lignocellulosic-reinforced epoxy composites, the present results show a comparable level of performance in terms of flexural strength. The values obtained here (44–54 MPa across RF, RG and RM) fall within the range reported for other short-fiber and particulate epoxy systems. Sarmin et al. [5] reported a flexural strength of 45.11 MPa for date palm fiber/chitosan particulate bio-epoxy composites at 20 wt.% filler loading, matching the reinforcement content used in this study. Kolawole et al. [26] reported a maximum flexural strength of 42.92 MPa for particulate date palm pit/epoxy composites at 10 wt.% filler loading, a lower reinforcement content than used here. Ghori et al. [27] reported a flexural strength of 64 MPa for alkalized date palm/kenaf hybrid epoxy composites at higher fiber loading (70 wt.% kenaf). These values are comparable to, or moderately higher than, the strengths observed in this study.

The quantitative results in Table 4 align with the fractographic observations reported above. RG combines the lowest mean flexural strength and modulus with a fracture surface showing the most extensive interfacial damage. RF combines the highest modulus with a fracture surface indicating the greatest resistance to crack advance. RM combines the highest mean flexural strength with an intermediate-to-high modulus, matching the intermediate fracture behavior described for this family. This numerical correspondence reinforces, without statistically confirming, the mechanistic interpretation proposed above.

## 4. Conclusions

This study assessed, for the first time, the potential of *Washingtonia robusta* pruning residue as a particulate reinforcement for epoxy biocomposites, examining the effect of reinforcement granulometry, fine, coarse and mixed fractions, on flexural stiffness, strength, damage tolerance and fracture micromechanisms.

Under an extended-duration test designed to capture its viscoelastic response, the neat epoxy resin (RS) accommodated the applied flexural load through a non-monotonic, viscoelastic-dominated stress–deflection response. It remained intact throughout the observation window, in contrast with the standard ISO 178:2019 [10] fracture characterization performed on the reinforced systems.

The incorporation of *Washingtonia robusta* particles, regardless of granulometry, reduced the matrix’s deformation capacity and shifted the failure mode toward progressive, particle- and interface-driven fracture within a substantially narrower strain range. Granulometry produced two distinct and partly opposing descriptive trends, detailed in Section 3.2. RF showed the highest flexural modulus (≈15.8 GPa) and the most delayed fracture. RG showed the lowest modulus (≈14.2 GPa) and the earliest fracture. RM combined a modulus close to RF (≈15.4 GPa) with the widest deflection-to-fracture trend among the three families. None of these differences reached statistical significance (one-way ANOVA, *p* > 0.05 in all cases). These trends should therefore be interpreted as descriptive rather than confirmed effects.

These findings suggest that granulometric control is a practical and low-cost lever for tailoring the flexural performance of natural particle epoxy composites. Particle shape and aspect ratio may also contribute to the observed effects and the results should therefore be interpreted as arising from granulometry as a whole rather than particle size in isolation. From an applied standpoint, the results support the potential of *Washingtonia robusta* urban pruning residue, currently disposed of without further use, as a locally sourced, unmodified candidate reinforcement for semi-structural epoxy applications. This waste valorization pathway represents a potential contribution to the broader effort to replace petroleum-derived and synthetic reinforcements with renewable alternatives. However, no lifecycle assessment, energy analysis, or carbon footprint calculation was performed in this study. Any environmental or low-carbon-footprint benefit should therefore be regarded as a potential advantage rather than an experimentally established one.

This study was limited in scope to flexural characterization and fractographic analysis; evaluation of tensile strength, impact resistance, moisture absorption, and long-term durability was not performed and remains necessary for a complete mechanical assessment of this reinforcement. Future work should address chemical or physical surface treatment of the reinforcement to further improve interfacial adhesion, particularly for the coarse fraction. Characterization should also be extended to tensile and impact loading, moisture absorption, and long-term durability to more fully establish the suitability of this reinforcement for structural applications.

## Figures and Tables

**Table 1:** Physicochemical properties of hardener component (manufacturer’s technical data sheet).

Property	Value	Test Method
Total anime value, TAV (mg KOH/g)	300–350	KD-AS-201
Viscosity at 25 °C (cP)	80–200	KD-AS-005
Color (Gardner)	80 max.	KD-AS-025
Recommended usage level (phr)	45–50	–

**Table 2:** Curing characteristics of epoxy resin–hardener system (YD-114F/KH-813).

Property	Value
Gel time (150 g scale)	59 min
Exothermic peak temperature	139 °C
Mixed-system viscosity (cP)	380

**Table 3:** Particle size fractions of *Washingtonia robusta* reinforcement.

Fraction	*n*	Average Size—40× (µm)	Average Size—300× (µm)
Fine (RF)	42	225 ± 41.0	27.8 ± 13.0
Coarse (RG)	16	505 ± 96.0	26.5 ± 14.6

**Table 4:** One-way ANOVA results (RF vs. RG vs. RM).

Variable	RF (Mean ± SD)	RG (Mean ± SD)	RM (Mean ± SD)	F-Ratio	*p*-Value
Flexural strength (MPa)	51.87 ± 9.48	47.38 ± 6.62	54.48 ± 8.10	0.97	0.41
Flexural modulus (GPa)	15.80 ± 1.43	14.22 ± 0.90	15.36 ± 0.90	2.69	0.11
Deflection at fracture (mm)	12.80 ± 1.17	11.02 ± 0.65	11.44 ± 1.52	3.16	0.08

## Data Availability

The original contributions presented in this study are included in the article. Further inquiries can be directed to the corresponding authors.
